# Ethics of Identifying Individuals Involved in HIV Transmission Events by Phylogenetics in Molecular Surveillance

**DOI:** 10.1111/bioe.70011

**Published:** 2025-07-22

**Authors:** Francisca Faber, Lucie Abeler‐Dörner, Stephanie Johnson, Tess Johnson, Euzebiusz Jamrozik

**Affiliations:** ^1^ Division of Neuropathology and Neurochemistry, Department of Neurology Medical University of Vienna Vienna Austria; ^2^ Ethox Centre and Pandemic Sciences Institute University of Oxford Oxford UK; ^3^ Royal Melbourne Hospital Department of Medicine University of Melbourne Melbourne Victoria Australia; ^4^ Monash Bioethics Centre, Monash University Melbourne Victoria Australia

**Keywords:** ethics, HIV, molecular surveillance, phylogenetics, risk–benefit analysis

## Abstract

Molecular HIV surveillance, involving the collection and analysis of HIV genome sequences, has become an integral part of public health programmes in high‐income countries. By employing phylogenetic analysis, molecular HIV surveillance can identify individuals and their positions within networks of HIV transmission. While the primary aim of molecular surveillance is to yield public health benefits, such as linking people to care and reducing transmission, it also poses risks and potential infringements on individual privacy and liberty. This paper examines the ethical implications of using phylogenetics to identify individuals involved in multiple transmission events in high‐income countries. Although public health responses tailored to such individuals can significantly reduce further transmission, these individuals often face multiple intersecting vulnerabilities and bear the greatest risks associated with molecular surveillance. We analyze the risks related to privacy, stigma, mistrust, criminalization, and liberty infringements, alongside the benefits of preventing further transmission and increasing healthcare engagement for people living with HIV. We conclude by outlining plausible and ethically acceptable policy options for molecular surveillance practice.

## 1 Introduction

Human immunodeficiency virus (HIV) is one of the fastest mutating viruses [1], and the accumulation of mutations over time results in unique HIV genomes between people living with HIV (PLHIV) [2]. Phylogenetics is the study of evolutionary relationships based on the similarities and differences in genetic material across biological entities. It allows discernment of relationships between HIV genomes and inference (with increasingly high certitude) of the directionality of evolutionary relationships and thus likely viral transmission between individuals [3].

In most high‐income countries, upon HIV diagnosis or initiation of antiretroviral treatment (ART), viral samples are sent for HIV genotyping and/or sequencing as part of standard medical practice. In many jurisdictions, these sequences and secondary sequencing analyses are stored in databases and used for public health purposes without additional consent, containing powerful information regarding HIV transmission in a community [4, 5, 6].

Where phylogenetic analyses are conducted on viral genomic data to monitor HIV in the community, this is a form of molecular HIV surveillance (MHS). MHS that makes use of phylogenetic analysis is currently used in countries including the United States, Canada, Australia, China, and multiple countries in the EU [7]. In a study conducted in 2016 to assess MHS in the EU, 15 out of 21 countries studied reported the use of phylogenetic analysis for surveillance of HIV transmission at a national level [7]. The usage of phylogenetics in MHS transmission is being scaled up around the globe [8].

In public health practice, HIV sequence data are rarely analysed alone. They are usually linked to other forms of data, such as sampling dates, information from contact tracing, and medical records. Integrating different data sets enhances the utility of data, by, for example, increasing the accuracy of source attribution [9]. The more data that is used, the more identifiable individuals within the data set become.

MHS in public health practice primarily aims to understand transmission patterns and is typically linked to public health actions that reduce HIV transmission. In some contexts, one set of public health actions will involve linking individuals to care. While linking people to care is generally noncoercive and intended to improve health outcomes, the use of phylogenetics to analyze HIV networks, and its potential to identify individual transmission events when combined with other data sets, raises significant ethical concerns [10]. Therefore, ethical analysis is important and timely, particularly given that this is already occurring within public health agencies [11].

In light of these concerns, this paper will explore the use of phylogenetics in public health, compare it with traditional contact tracing, examine ethical considerations including risks and benefits to individuals that MHS can identify to have high transmission numbers, and propose context‐sensitive policies developed in collaboration with PLHIV. These should include selective analysis approaches (e.g., population‐level vs. individual level), adequate data protection, limited transparency around source attribution, and minimally‐ or noncoercive public health responses.

### 1.1 Details on Phylogenetics

**Figure 1:**
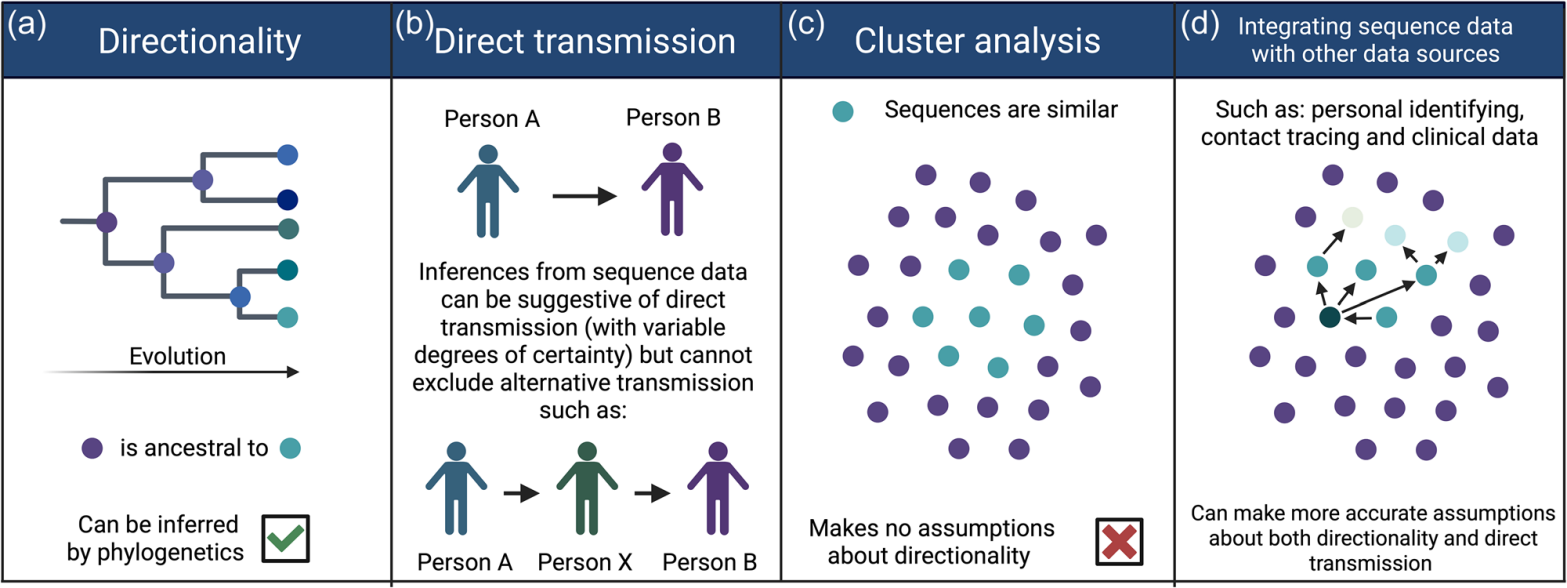
Different terminology concerning phylogenetics in molecular surveillance. (a) Directionality is visualized with phylogenetic trees and gives information on the ancestry of related viral genomes. Directionality can be inferred by phylogenetics. (b) Direct transmission is the transmission of virus from person A directly to person B. Direct transmission cannot be inferred by phylogenetics with certainty, due to sampling gaps, such as person X being an intermediate in a transmission pair. (c) Cluster analysis reveals similar sequences but makes no assumptions about directionality or direct transmission. (d) Integrating phylogenetic data with other data sources can make increasingly reliable assumptions on directionality and direct transmission. Created with BioRender.com.

While sophisticated phylogenetic analyses can distinguish *directionality* with a high degree of certainty, HIV sequence data alone cannot provide definitive evidence that *direct transmission* has occurred between two individuals [6, 12, 13]. Directionality and direct transmission are frequently conflated [6]. Directionality reveals which viral sequence is ancestral in the evolutionary chain, thereby identifying the chronologically older variant (Figure 1a) [14]. Direct transmission aims to elucidate if sample A is the direct source of sample B, hence whether the virus was transmitted from person A directly to person B. Although improvements have been made in inferring directionality in transmission pairs, this is not equivalent to establishing direct transmission with certainty. This is partly due to potential sampling gaps, for example, there can be an unsampled individual (person X), who is an intermediary in the transmission between person A and person B, or the common source of infection (Figure 1b) [6, 9, 14].

An alternate method for using HIV sequences to extract information about the relationships between viruses is through *cluster analysis*. In this method, genetically highly similar sequences are identified as a cluster and can signify rapid transmission if a cluster contains many recent infections [15]. Clustering approaches make no statement about the directionality of transmission (Figure 1c). To further avoid attributing transmission to specific individuals, clusters can include a minimum threshold number of individuals. However, when cluster analysis is integrated with other data sources, such as contact tracing and clinical data, directionality and direct transmission could potentially be deduced (Figure 1d).

Although the possibility of inferring direct transmission with phylogenetics alone is disputed, some argue that with sufficient data integration, direct transmission can undoubtedly be inferred [16]. This integrative approach is increasingly applied in real‐world public health settings to guide targeted interventions. One illustrative example comes from Canada, where MHS has been used in combination with other tools to respond to potential outbreaks.

### 1.2 Case Studies of Molecular Surveillance Targeting Individuals

In a study by Poon et al. (2016), viral data were reanalysed by a monitoring system integrated into the British Columbia drug treatment database to detect clusters with short phylogenetic distances upon deposition of new HIV genotypes. When a rapidly growing cluster of drug‐resistant HIV among a community of men who have sex with men (MSM) was identified, these cases were reported to public health authorities who offered enhanced follow‐up, consisting of improving individuals’ linkage to care and conducting additional contact tracing interviews. Seven out of nine individuals contacted consented to participate in the enhanced follow‐up, leading to the identification of additional sexual contacts.

One of these additional contacts was found to be HIV‐positive, disengaged from care, and involved in high‐risk behaviors, suggesting he may have been involved in transmission. This individual was contacted and re‐engaged in care [17]. This case study demonstrates how public health services can identify and contact individuals through phylogenetic data.^1^

In this study, the phylogenetic cluster was defined at a minimum size of five individuals to prevent source attribution. However, due to the characteristics of the additionally identified contact, it was suggested he was involved in transmission. So, although the use of larger clusters may be a safeguard to reduce the ascription of transmission to an individual, individuals are nevertheless identifiable as likely sources when HIV sequence data is used in combination with other types of data.

Another exemplary study is that of Little et al. (2014), who analyzed the viral sequences and sexual networks of recently infected individuals in San Diego. They conducted a retrospective analysis that showed that targeted ART to individuals with a high transmission network score would have significantly (*p* < 0.05) reduced HIV transmission in that network. They concluded that identifying and targeting high‐risk individuals can effectively prevent HIV transmission [2].

Together, these studies illustrate the potential of molecular surveillance to inform targeted interventions. As phylogenetic methods become more advanced, the ability to identify individuals as potential sources of HIV transmission is likely to become more reliable and widespread. Therefore, models of ethical best practice for such data in public health are important.

### 1.3 Comparison Between Molecular Surveillance Using Phylogenetics and Traditional Contact Tracing

To assess the ethical issues related to MHS using phylogenetics, it is crucial to consider how this approach differs from traditional HIV surveillance methods like contact tracing (Table 1). With this comparison, we can assess what marginal benefit there is to phylogenetics, as well as any additional risks or harms that might counterbalance this benefit.

**Table 1:** Differences of contact tracing, phylogenetics and their combination in HIV molecular surveillance. People living with HIV (PLHIV).

Contact tracing	Phylogenetics	Contact tracing combined with phylogenetics
Fragmented network(s)	Integrated network(s)	Most comprehensive network coverage
Dependent on information disclosed by interviewee	Can bypass willingness of individual to disclose certain details	Can rely on both disclosed and/or non‐disclosed information
Does not aim to establish directionality	Assesses directionality	Can increase directionality accuracy by combining chronological events and social relationships from contact tracing with the ancestral viral relationships in phylogenetics
Gathers knowledge of both PLHIV and people at‐risk	Can only be conducted with information from PLHIV	Gathers knowledge of both PLHIV and people at‐risk
Detects transmission at later stage	Detects transmission at earlier stage	Detect transmission early and provides the best social indication for intervention
Provides information about potential transmission between individuals	Can highlight gaps in contact tracing efforts	Provides the fullest coverage of transmission dynamics
Less scalable (due to intensity of interviews)	More scalable	Moderately scalable ‐ requires coordination between staff with multiple areas of expertise

**Figure 2:**
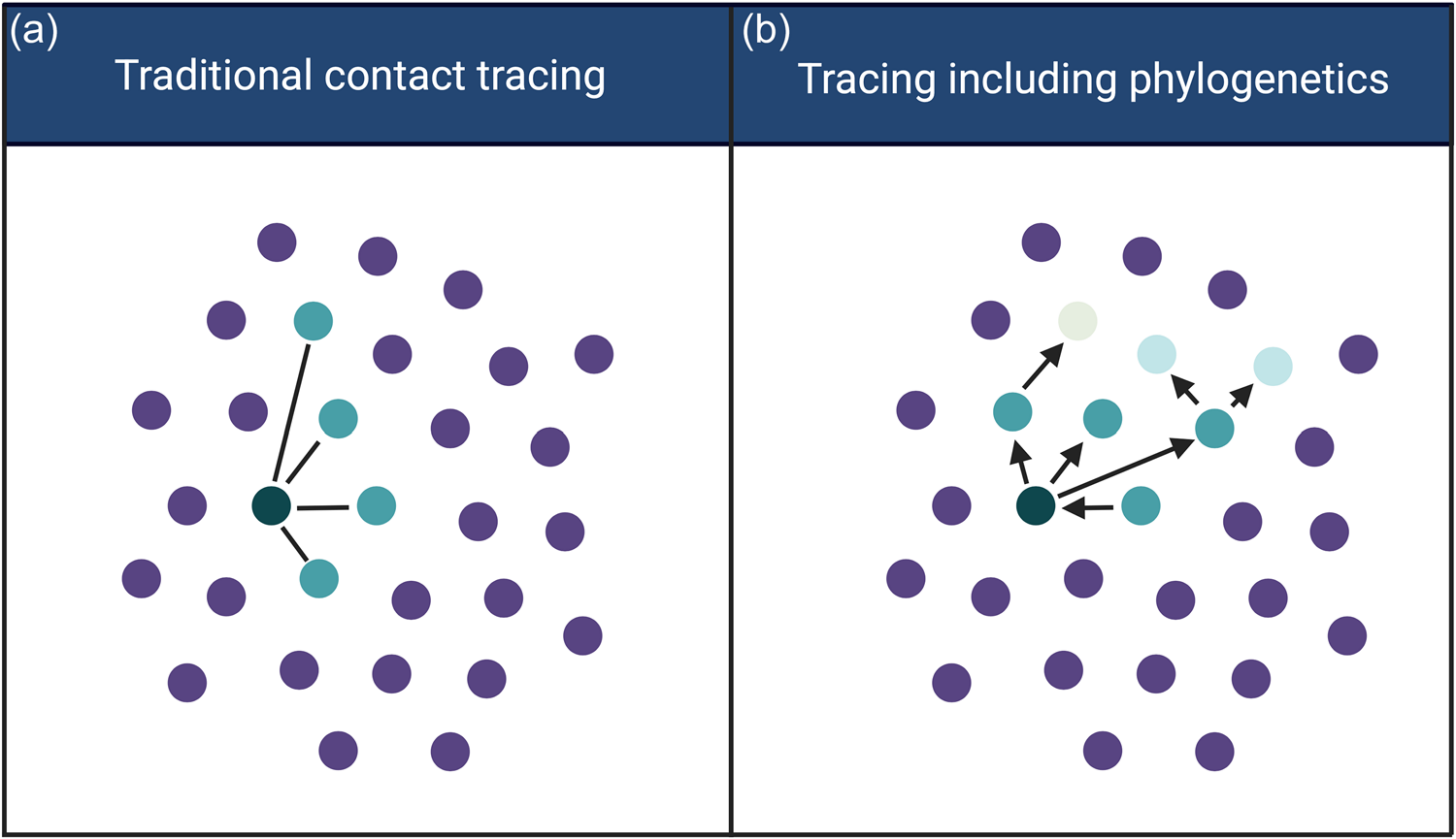
Traditional contact tracing and tracing including phylogenetic analysis. (a) In traditional contact tracing, networks are based on interviews that reveal the secual history of indivduals involved. (b) When phylogenetic analysis are combined with contact tracing data, more complex networks including directionality can be reconstructed based on molecular similarities. Molecular data can include individuals in transmission networks (who were not identified in contact tracing), exclude individuals from transmission networks (e.g., two or more PLHIV with reported sexual contacts where molecular data demonstrates that sequences are not linked), or identify individuals as likely intermediary points of transmission between two other PLHIV. Created with BioRender.com.

First, as contact tracing relies solely on disclosed information, it may provide a more fragmented view of a transmission network (Figure 2a). In contrast, phylogenetics can provide a more integrated perspective, capable of connecting or ruling out many viral relationships at once and revealing more complex transmission patterns (Figure 2b). Phylogenetics can act both as a proxy for social clustering and as a tool for capturing broader, population‐level structures [18]. It can identify links between otherwise disconnected clusters, especially when partners are not disclosed during interviews (or where individuals’ knowledge of their sexual networks is complete. For instance, an individual not reporting sexual behaviour with other men can be connected to MSM clusters through phylogenetic analysis. This implies that, unlike contact tracing which relies on information voluntarily disclosed, MHS can bypass this willingness [19]. This can increase the accuracy of transmission network reconstruction and potentially inform public health measures, but also raises ethical concerns.

Second, traditional contact tracing and phylogenetics differ in their capabilities to assess directionality. Although phylogenetics cannot prove direct transmission with certainty, it can give probabilities for directionality and thus indicate who might have transmitted HIV to whom, whereas standard contact tracing provides minimal information on the directionality of transmission (e.g., because contact tracing data cannot determine whether HIV sequences are closely genetically related) [2]. Combining chronological events from contact tracing with the ancestral relationships from phylogenetics can enhance the accuracy of determining transmission directionality.

Third, MHS can only be conducted on PLHIV whose HIV samples are in the surveillance database, although the information obtained from these analyses can also have an impact on sexual contacts who are HIV‐negative. On the other hand, contact tracing provides information both on individuals diagnosed with HIV and their close contacts (who may not necessarily be living with HIV or not be aware of their status). Among other things, this means that contact tracing involves privacy infringements for more individuals, while the privacy infringements of MHS are more concentrated among PLHIV.

Four, MHS can detect growing clusters at an earlier stage than partner services [20]. Additionally, because individuals whose HIV sequence is not linked to any other sequences in the community may have acquired HIV from undiagnosed individuals (whose sequences are not yet held in MHS databases), MHS can inform estimates regarding the number of undiagnosed individuals, highlighting gaps in contact tracing efforts [21, 22].

Fifth, unlike contact tracing, MHS does not require interviews, making it more scalable and less time‐consuming. This might make MHS more efficient and implies it can provide complex information on large groups of individuals (that could be updated over time as new sequences are added to the database). One key difference is that MHS will not include data on (undiagnosed and/or uninfected) contacts of PLHIV—meaning that traditional contact tracing (such as partner services interviews) will not be entirely replaced by MHS. However, MHS might reveal transmission dynamics not identified by contact tracing, such as cryptic transmissions [23]. In turn, this allows MHS to support the efficiency of contract tracing, as it could be focused where molecular data suggest that recent transmissions are most concentrated or where the sources of a case or cluster are unclear.

Sixth, the laws and regulations that govern the use of traditional contact tracing and MHS at present differ significantly. Contact tracing is a recognised public health activity, falling under the remit of health protection teams, with the expectation that it involves clinical interactions with patients. MHS is traditionally a research and surveillance activity, typically undertaken by public health experts, sometimes in collaboration with researchers. It is only publishable when the data is pseudonymised, with no expectation of identifying or interacting with individuals. This distinction changes the regulatory and ethical norms and obligations of those involved in undertaking the two practices.

Due to the differences between traditional contact tracing and phylogenetics as just outlined, the use of phylogenetics requires its own distinct ethical justification when used in public health programmes.

### 1.4 Ethical Justification of Phylogenetics

While one purpose of MHS is the identification of gaps in HIV interventions at the level of populations or subpopulations (rather than the identification of individuals) [24], some public health agencies are already using or planning to use phylogenetics to identify and perhaps contact individuals to link them to care to prevent further infections [2, 17]. The ethical acceptability of using HIV phylogenetics for this purpose depends in part on the additional efficacy.

Additionally, an approach to the ethical justification of MHS for purposes of source attribution might be to require that there are benefits on the individual level. For example, Molldrem and Smith (2020) argue that ‘[a]rticulating the benefits of MHS [molecular HIV surveillance] and CDR [cluster detection and response] to individual people—not only for “communities” or “the public”—is necessary for the continued justification of these interventions’ [25].

**Figure 3:**
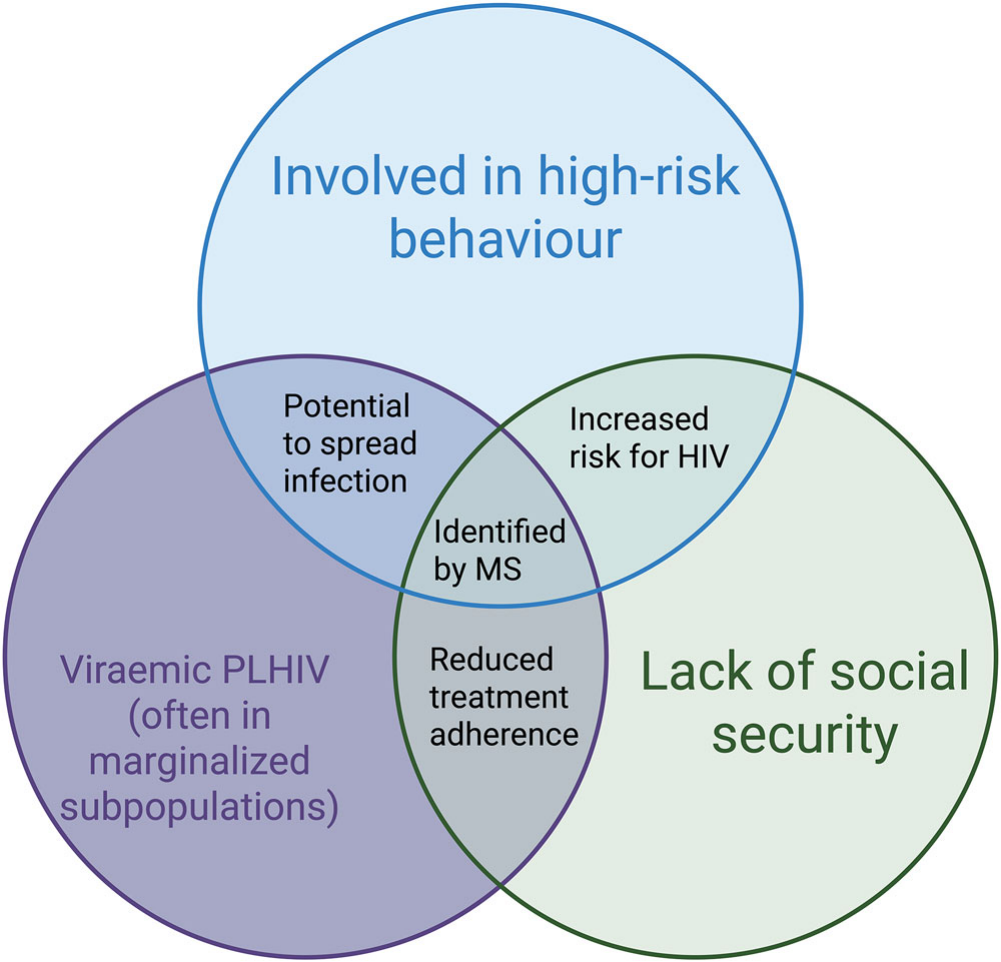
Intersecting vulnerabilities of individuals identified by molecular surveillance (MHS). Created with BioRender.com.

One reason for this position is that there may be significant harms (and relatively few benefits) for individuals whose HIV sequence data are used, often without consent, to produce public health benefits. Additionally, the individuals identified by MHS to have high transmission numbers often have multiple intersecting vulnerabilities and will bear the greatest burdens related to MHS, due to associations between HIV status and other factors and identities (Figure 3). In high‐income countries, HIV epidemics are often concentrated in certain subpopulations, such as MSM, people who inject drugs, prisoners, sex workers, transgender people, and migrants [26]. Among PLHIV, treatment adherence is influenced by aspects of social security, such as housing, understanding of HIV, and trust in the healthcare system [27]. Focussing phylogenetics on identification at the individual level would likely disproportionately involve the most vulnerable people of already marginalised groups affected by HIV. However, other individuals may benefit, such as people who are HIV‐negative but are at risk of acquiring HIV from their contacts. These individuals will likely also belong to vulnerable populations and will thus be protected by these MHS practices. Below, we develop a risk/benefit analysis of the effects experienced by the individuals identified by MHS to be involved in transmission events (Table 2).

**Table 2:** Risks and benefits for individuals identified by molecular HIV surveillance aimed at source attribution.

Risks	Benefits
Violation of privacy	Help to relink to care for those previously diagnosed
Liberty infringements	Reducing AIDS
Increased stigma	Accurate targeting of resource‐intense approaches
Violation of trust	
Criminalization	

### 1.5 Benefits for Identified Individuals

#### 1.5.1 Engaging Patients in Healthcare

For PLHIV who are not currently undergoing ART or are encountering challenges with treatment adherence, MHS can be more effective than current public health practices in identifying and supporting them [2]. This could result in a reduction of progression from HIV to AIDS and a more accurate allocation of resources. For instance, personalised care strategies, such as intensified adherence counselling sessions or discussions with healthcare professionals about treatment aversions and perceived risks, can increase adherence and reduce individual risks of AIDS. MHS could ensure that these resource‐intense approaches are directed to where they are most needed, that is, where increased adherence may have the most benefits for individuals and/or public health [5].

#### 1.5.2 Benefits for Individuals Protected From Future Infection

While the risk–benefit analysis focuses on PLHIV whose data are used in MHS, it is important to note that benefits will likely be largest for those whose future HIV acquisition is prevented, rather than those identified as transmission sources by MHS. By enabling the identification of individuals more likely to transmit HIV, MHS can help concentrate public health resources where the probability of new infections is highest, thereby reducing the incidence of HIV [28, 29, 30]. However, individuals in both groups may be more likely to belong to populations of intersecting vulnerabilities—suggesting that both the benefits and the burdens of MHS will be concentrated among worse‐off individuals or groups. Therefore, justice considerations regarding the use of MHS require careful consideration.

### 1.6 Risks for Identified Individuals

#### 1.6.1 Violation of Privacy

Contact tracing requires either implicit or explicit consent from interviewees, who thereby accept the privacy infringements involved in the disclosure of personal information (although they may also reveal private information about other non‐consenting people, such as their HIV status, sexual practices, etc.) In contrast, MHS is often conducted without the knowledge or consent of the individuals whose data are used and provides information about much larger contact networks.

In any case, the number of HIV transmission events linked to a particular person and the involvement in transmission chains is highly sensitive information (whether such data are collected as part of MHS, contact tracing, or both). When data sets are integrated and include sequence data or assessments of source attribution or direct transmission, this arguably goes beyond the privacy infringements involved in standard contact tracing practice and therefore may require a more cautious approach [30].

#### 1.6.2 Liberty Infringements

HIV public health programmes potentially involve significant infringements of individual liberty. This may occur via (i) pressure to adhere to therapy (from clinicians or public health agencies), (ii) legal restrictions on behaviour (e.g., curfews or condom use), (iii) directly administered therapy, and/or (iv) other treatment mandates including the use of injectable antiretrovirals. MHS techniques possess considerable potential to inform these less frequent but more extreme public health measures.

On the one hand, the use of MHS in deciding where to target such coercive measures might make them more proportionate insofar as they are focused where the risks of a person transmitting to others are highest. On the other hand, such approaches may exacerbate injustice because individuals involved in multiple transmission events often have numerous vulnerabilities, and coercion may therefore be concentrated among the most marginalised and vulnerable PLHIV. Furthermore, this could increase mistrust in the healthcare system precisely among the groups where trust is especially absent and fragile.

These complexities regarding coercive measures may come to a head if individuals identified via MHS decline ART. While patients in liberal democracies maintain the right to refuse medical interventions, these rights are balanced with the imperative to prevent HIV transmission to others (i.e., with the rights of others not to be harmed). Indeed, public authorities may be considered to have an obligation to prevent harm to others via coercing those with high transmission rates who decline ART under other preventive measures. Yet, individuals’ ART non‐adherence is impacted by multiple factors, including distrust in medication, not feeling sick, stigmas associated with ART, challenges in adhering to daily medication regimens due to social or occupational obligations, and side effects [31, 32].

Coercive measures for HIV‐positive individuals can consist of pressure to adhere to ART and other legal restrictions. Such measures are used as a last resort in certain countries, including Canada and Australia, where an individual perceived as a health risk by authorities, can be legally mandated to adhere to a curfew, undergo counselling, use condoms, or undergo ART [33]. Other potential measures include directly‐administered ART (DAART), which involves outreach workers observing the administration of oral ART. Research on the superiority of directly‐administered ART (DAART) has already been conducted [34]. Additionally, injectable ARTs that require injections every 2 months could be used [35], which may be perceived to involve an additional infringement of bodily integrity, as these medicines are injected by a healthcare worker rather than administered by the patient themselves. While such interventions do not require molecular data (contact tracing and other nonmolecular data have been used to justify such measures), MHS could enable health agencies to identify individuals at higher risk of transmitting to others with greater accuracy and could provide a stronger justification for coercive action.

#### 1.6.3 Increased Stigma

Attributing transmission to individuals can increase the perception among members of the public that individuals are ‘vectors of disease’ and responsible for certain cases of transmission [36]. This could increase stigma, as well as invoke feelings of guilt about transmitting to others and have negative consequences on social lives, mental health, and trust in the healthcare system.

Traditional contact tracing aims to alert those who might be HIV‐positive or at risk of infection. Additionally, in combination with other types of data, it is used to identify individuals who are considered to be ‘high risk’ or more likely to transmit to others [37, 38]. The directionality attributions possible through phylogenetics in MHS could lead to public health agencies identifying and responsibilising ‘high risk’ individuals with a much higher degree of accuracy. This individualised view of transmission potentially shifts focus away from improving the structural factors that contribute to HIV risk.

#### 1.6.4 Violation of Trust

Lack of informed consent for using sequence data from HIV tests collected for clinical purposes may lead to mistrust in health or government institutions among PLHIV. Unlike contact tracing, MHS techniques circumvent the willingness of individuals to disclose information [19]. Traditional contact tracing relies on the input of the involved individual, whilst MHS can identify contacts (involving transmission) that individuals did not disclose in contact tracing interviews. As mentioned by Sandset (2020) ‘molecular transmission tracing (…)—offer[s] ways of circumventing the subject's own willingness to disclose (…) with whom they have sex’. This can become especially sensitive in the context of identifying someone as a member of certain subpopulations, for instance, when MHS identifies a heterosexual‐identifying individual to be involved in MSM transmission networks. Initiating contact about such findings could violate trust. This can lead to additional harms, such as causing individuals to avoid engagement with healthcare. Such effects may be amplified in communities that are already disproportionality affected by HIV and have experienced historical breaches of trust by members of the medical profession, either as a population or as individuals. In line with these concerns, interviews with HIV stakeholders all supported the idea that PLHIV should be informed of how their data is used [39].

#### 1.6.5 Criminalisation

Another concern related to source attribution through MHS is the increased prosecution of criminal cases of HIV transmission. When public health agencies hold phylogenetic data on transmission events and personally identifiable data, they can be subpoenaed by legal authorities for criminal HIV transmission cases, despite ethical norms that surveillance data should not be shared for non‐health purposes [8]. Although MHS, even when combined with other databases, cannot give definite proof of direct transmission events, such data could, and already has been, used for this purpose in criminal investigations [40].

## 2 Policy Options

**Figure 4:**
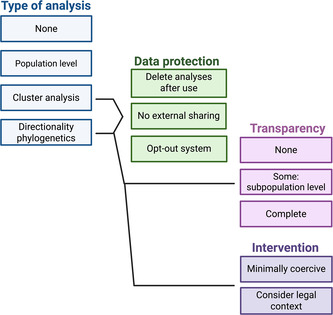
Decision tree for different policy options for the implementation of HIV molecular surveillance, ranging from not analysing molecular data to using phylogenetics to identify directionality. Cluster analysis and phylogenetics to identify directionality arguably warrant additional data protection measures. Public health agencies must also determine the appropriate intervention and level of transparency regarding the use of molecular data and the results of phylogenetic analyses. Created with BioRender.com.

Policymakers considering the use of phylogenetics in MHS face multiple decisions regarding (i) the type of analysis to employ, (ii) data protection measures, (iii) transparency regarding source attribution, and (iv) appropriate public health intervention (Figure 4). Each of these aspects requires evaluation to determine an appropriate approach for MHS in any given context.

### 2.1 Type of Analysis

Options for the use of molecular HIV data by public health agencies include (i) abstaining from MHS, (ii) population‐level summaries, (iii) cluster analysis, or (iv) phylogenetics where directionality is assessed. Complete abstention from MHS is improbable in high‐income countries. As HIV sequencing and sharing of sequences with public health agencies becomes routine, these data will likely be used in at least some way to inform public health practice.

In some contexts, it may be ethically preferable to conduct only population‐level assessments of HIV diversity and ART resistance. This might be preferred in regions where PLHIV endure significant stigma and marginalisation and there is mistrust in public health agencies. More individual‐level MHS analysis might further damage the trust of PLHIV and, among other negative effects, contribute to disengagement from care. On the other hand, in some liberal democracies with less HIV stigma and adequate trust in healthcare systems, employing phylogenetics for cluster detection might be more acceptable. Cluster analysis typically creates less sensitive data as it does not assess the directionality of transmission. However, it can still provide indications of directionality in combination with other data. For example, in the case study by Poon et al. (2016), analysis was limited to clusters over five people to prevent personal identification, yet an individual was still identifiable as involved in transmission.

Compared with cluster analysis, other phylogenetic analyses may be more likely to focus on specific individuals to increase engagement with treatment. This approach might be more acceptable to PLHIV in contexts where there is a high level of trust in public health systems, low levels of stigma, and a primarily noncoercive approach to public health practice.

The appropriate type of analysis thus depends on the local context and how a proposed use of MHS affects the potential benefits and risks of the public and the individuals involved. Context‐specific research to estimate such outcomes should involve community engagement and collaboration with groups of PLHIV, particularly those with relevant vulnerabilities. It should thoroughly consider all potential risks and benefits that can affect individuals including those outlined above. Assessing these risks and benefits both before and during MHS practices is essential to maintaining trust in healthcare and preventing people from avoiding healthcare services.

### 2.2 Data Collection and Protection

Special consideration for handling and safeguarding data is imperative given the sensitive nature of MHS data. In addition to the use of secure databases, such protections could include (i) deletion of phylogenetic analyses immediately after use; (ii) refraining from sharing deep‐sequencing data and the results of source attribution analyses with external parties, including those identified in the data and law enforcement agencies; and/or (iii) permitting individuals to opt out of MHS without changes to their access to care.

First, phylogenetics involves the generation of phylogenies, that is, maps of evolutionary relationships between HIV samples. One data protection measure would be to ensure that such phylogenies are temporary and used only for the duration of the necessary analysis. This can safeguard especially sensitive information by ensuring it is not stored for prolonged periods. Importantly, this does not include deletion of the sequence data itself, only of the analytical outputs, such as phylogenies. Further outputs can be generated as needed when new sequences are added and/or new analyses are required for public health action.

Second, sharing sequence data, in particular deep‐sequencing data, and results of analyses should be minimised. While some individuals might wish to receive information regarding the connection of their viral sequence within the phylogenetic tree, and/or assessments of the likely source(s) of their infection, it may often be considered unethical to disclose these results to individuals because such data are inherently relational and may involve unacceptable infringements of the privacy of others. Additionally, sharing data with third parties should be limited as much as possible. However, since data can be subpoenaed for criminal cases, there may be a need to protect the privacy of individual data and the appropriate activities of researchers or public health agencies via legislation and/or regulatory norms.

Third, a possible strategy for more ethical data collection is an opt‐out policy regarding the use of genotyping data in MHS. This could mitigate concerns regarding unwanted and uninformed use of personal data. However, there may arise issues with how many individuals would opt out, especially in vulnerable populations. To demonstrate, Buchbinder et al. (2022) conducted interviews with previously incarcerated PLHIV and noted that all participants stated that consent should be obtained for the use of HIV data, however only half of the participants would give this consent if asked [41]. An opt‐out system thus has the risk of missing the subpopulations where HIV assistance is most needed. Although how much this would undermine the public health benefits of MHS in specific contexts is often unknown, therefore more research is needed on this topic as also suggested and elaborated on by Molldrem et al. (2023) [42]. In any case, permitting an opt‐out might, help foster trust in healthcare systems. As mentioned by Molldrem et al. (2023) ‘doing the hard work of securing the buy‐in of people living with HIV to participate in public health programs offers an opportunity to build trust and strengthen health systems by affording individuals more chances to engage with how they receive care and other supportive services’ [42]. They additionally provide four proposals on potential opt‐out scenarios, such as creating a dynamic consent approach [42].

In line with these efforts, parallel initiatives aimed at educational outreach and community engagement as illustrated by Dennis et al. (2023) regarding MHS could be implemented and focus on affected subpopulations [43]. These efforts have the potential to enhance public understanding and acceptance of MHS, thereby decreasing the number of people opting out. In this manner, the underlying issue of distrust in MHS can be addressed, while simultaneously improving the ethical acceptability of the use of molecular data.

### 2.3 Transparency

An important aspect of an MHS approach aimed at tracing individuals is the extent to which public health agencies should be transparent about the *use* of such data, the extent to which results of analyses should be disclosed, and to whom such data should be disclosed, if at all.^2^ At the general community level, public health agencies should arguably be transparent about the use of MHS, including the types of data used, the goals of such programmes, and mechanisms of oversight. This transparency would enable community engagement and education efforts to inform PLHIV of their use of molecular data in MHS. The approach to such engagement can be informed by previous studies [44], with one example being the participatory praxis approach [45].

Questions about transparency regarding the *results* of phylogenetic source attribution analyses may be more complex. Such results involve extremely sensitive information that should not be disclosed to the public, especially in any way where individuals could be potentially re‐identified (e.g., based on associated data such as age, gender, postcode, etc.). Even the publication of summary data may have negative effects, for example by contributing to stigma for groups identified as involved in transmission clusters or events. Public health agencies should therefore carefully consider any plan to publish such data.

There are also important ethical questions regarding the disclosure of the results of source attribution analyses to the individuals involved. Non‐transparent approaches could involve contacting individuals identified as the source of transmission without informing them of these results and instead under the pretext of routine check‐ups or because of other risk factors for transmission (such as treatment non‐adherence identified through pharmacy records). This may avoid causing feelings of guilt or other burdens. However, such approaches involve deception, either at the level of staff who contact PLHIV (if staff are informed of results) or at the level of public health agencies (if individual staff were blinded to the results of source attribution).

More transparent approaches could involve informing regarding the number of transmission events to which they have been linked. The extent to which this would produce benefits via improved engagement with treatment or harms related to feelings of guilt and/or stigmatisation is uncertain, and it would involve significant privacy infringements for the people identified as sources of transmission.

Given these tradeoffs between privacy, transparency, and deception, as well as uncertainty about risks and benefits, determination of the optimal approach necessitates further research conducted in close collaboration with PLHIV to ascertain the acceptability of different options. While some form of transparency may foster trust between PLHIV and public health workers, complete transparency is likely not desirable as the privacy infringements would be too detrimental.

#### 2.3.1 Appropriate Public Health Intervention

Lastly, an essential aspect of ethical MHS is ensuring that public health interventions are appropriate and minimally coercive. Interventions should focus on reconnecting individuals to care rather than implementing punitive measures or assigning blame, this aligns with the goal of public health of helping individuals to the care they need and deserve. Additionally, interventions must consider the legal context. Especially in regions where homosexuality is criminalised or where there are specific laws regarding HIV transmission, interventions aimed at the individual level must be avoided or navigated with caution, ensuring that the measures taken are ethical and effective within the given legal environment.

## 3 Conclusion

Phylogenetic analyses are becoming more accurate, and public health agencies are increasingly using these methods as part of MHS. As these approaches are adopted, they are likely to identify individuals with multiple vulnerabilities, and it is important to consider the effects MHS will have on these individuals and the wider community. We argue that a range of benefits and risks might arise from MHS for individuals identified as transmission sources, such as allocation of resources, health considerations, stigma, trust, privacy, liberty and criminalization. This analysis can inform future empirical research aimed at evaluating risk/benefit ratios among affected individuals as well as policy deliberations regarding the ethical acceptability of different MHS approaches. Policy decisions should consider the appropriateness of data analysis methods, data protection measures, and approaches to the disclosure of source attribution analyses (if any). Additionally, policy development should take into account the local context and the risks and benefits for individuals identified through MS and assess whether the anticipated additional advantages justify the potential negative consequences of its use. Limitations of this analysis include that, although HIV genomic data is increasingly used in surveillance, there are few other published reports of public health applications of these approaches. Although such reports require careful efforts to protect the privacy of PLHIV, there is arguably an ethical case for wider reporting of public health applications to promote transparency in the use of genomic surveillance for HIV. These could also inform future research on ethical policy for the use of phylogenetics.

## Data Availability

Data sharing is not applicable to this article as no data sets were generated or analysed during the current study.

## Endnotes

1 It is unclear whether the viral sequence and/or degree of linkage with positive cases of the identified individual was already on file. He was known to be HIV‐positive and disengaged from care, however, he was identified through follow‐up contact tracing, and not phylogenetic analysis.

2 It is unlikely that it would be widely considered ethically acceptable to disclose source attribution data to all individuals of the transmission events, given privacy infringements and potential harmful conflicts between individuals without any significant benefits.
